# Multisite Harmonization of Structural DTI Networks in Children: An A-CAP Study

**DOI:** 10.3389/fneur.2022.850642

**Published:** 2022-06-17

**Authors:** Adrian I. Onicas, Ashley L. Ware, Ashley D. Harris, Miriam H. Beauchamp, Christian Beaulieu, William Craig, Quynh Doan, Stephen B. Freedman, Bradley G. Goodyear, Roger Zemek, Keith Owen Yeates, Catherine Lebel

**Affiliations:** ^1^Department of Psychology, University of Calgary, Calgary, AB, Canada; ^2^MoMiLab Research Unit, IMT School for Advanced Studies Lucca, Lucca, Italy; ^3^Department of Neurology, University of Utah, Salt Lake City, UT, United States; ^4^Hotchkiss Brain Institute, University of Calgary, Calgary, AB, Canada; ^5^Alberta Children's Hospital Research Institute, University of Calgary, Calgary, AB, Canada; ^6^Department of Radiology, University of Calgary, Calgary, AB, Canada; ^7^Department of Psychology, University of Montreal and CHU Sainte-Justine Hospital Research Center, Montreal, QC, Canada; ^8^Department of Biomedical Engineering, University of Alberta, Edmonton, AB, Canada; ^9^University of Alberta and Stollery Children's Hospital, Edmonton, AB, Canada; ^10^Department of Pediatrics, British Columbia Children's Hospital Research Institute, University of British Columbia, Vancouver, BC, Canada; ^11^Departments of Pediatrics and Emergency Medicine, Alberta Children's Hospital Research Institute, Cumming School of Medicine, University of Calgary, Calgary, AB, Canada; ^12^Department of Pediatrics and Emergency Medicine, Children's Hospital of Eastern Ontario Research Institute, University of Ottawa, Ottawa, ON, Canada

**Keywords:** diffusion MRI, structural connectome, multisite harmonization, ComBat, graph theory, pediatric mild traumatic brain injury

## Abstract

The analysis of large, multisite neuroimaging datasets provides a promising means for robust characterization of brain networks that can reduce false positives and improve reproducibility. However, the use of different MRI scanners introduces variability to the data. Managing those sources of variability is increasingly important for the generation of accurate group-level inferences. ComBat is one of the most promising tools for multisite (multiscanner) harmonization of structural neuroimaging data, but no study has examined its application to graph theory metrics derived from the structural brain connectome. The present work evaluates the use of ComBat for multisite harmonization in the context of structural network analysis of diffusion-weighted scans from the Advancing Concussion Assessment in Pediatrics (A-CAP) study. Scans were acquired on six different scanners from 484 children aged 8.00–16.99 years [Mean = 12.37 ± 2.34 years; 289 (59.7%) Male] ~10 days following mild traumatic brain injury (*n* = 313) or orthopedic injury (*n* = 171). Whole brain deterministic diffusion tensor tractography was conducted and used to construct a 90 x 90 weighted (average fractional anisotropy) adjacency matrix for each scan. ComBat harmonization was applied separately at one of two different stages during data processing, either on the (i) weighted adjacency matrices (matrix harmonization) or (ii) global network metrics derived using unharmonized weighted adjacency matrices (parameter harmonization). Global network metrics based on unharmonized adjacency matrices and each harmonization approach were derived. Robust scanner effects were found for unharmonized metrics. Some scanner effects remained significant for matrix harmonized metrics, but effect sizes were less robust. Parameter harmonized metrics did not differ by scanner. Intraclass correlations (ICC) indicated good to excellent within-scanner consistency between metrics calculated before and after both harmonization approaches. Age correlated with unharmonized network metrics, but was more strongly correlated with network metrics based on both harmonization approaches. Parameter harmonization successfully controlled for scanner variability while preserving network topology and connectivity weights, indicating that harmonization of global network parameters based on unharmonized adjacency matrices may provide optimal results. The current work supports the use of ComBat for removing multiscanner effects on global network topology.

## 1. Introduction

Network neuroscience has become a popular approach to characterize brain structure *in vivo* in healthy and clinical populations (1–3). The structural connectome can be mapped using diffusion-weighted MRI (2, 4–6), a non-invasive technique that is sensitive to white matter microstructure (7).

Pediatric mild traumatic brain injury (mTBI) is a prevalent global public health concern (8–11) that is characterized by subtle and diffuse alterations in brain tissue [reviewed in (12, 13)]. The neurobiology of pediatric mTBI remains poorly understood [see (12)]. White matter microstructural alterations can occur after pediatric mTBI, and multiple studies have examined specific white matter tracts using diffusion tensor imaging [DTI; (13–15)]. Emerging evidence indicates that pediatric mTBI can alter global and regional brain networks (16–19). Thus, network neuroscience may be a potentially promising tool that could provide a robust characterization of network mechanisms involved in this important and highly prevalent neurological disorder.

Large, multisite neuroimaging studies of pediatric mTBI have become increasingly common to reduce false positive results from small samples, increase statistical power, and enhance reproducibility and generalizability of results (20, 21). For instance, the Advancing Concussion Assessment in Pediatrics (A-CAP) study (22) is the largest neuroimaging study of pediatric mTBI to date, with recruitment occurring at five children's hospitals across Canada including longitudinal MRI assessment using 6 different scanners. The A-CAP study has the potential to increase scientific and clinical knowledge about neurobiological outcomes in pediatric mTBI. However, using multiple MRI scanners introduces non-biological data variability due to different scanner systems, models, and sequence protocols, among other factors (23–26). Managing these non-biological sources of variability in multisite studies is increasingly important to generate accurate group-level inferences and enable detection of underlying biological phenomena (23).

ComBat is a widely used method for multisite (multiscanner) harmonization that originated from techniques used for genomics data (27). It is one of the most well-validated tools for multiscanner harmonization of structural neuroimaging data that makes no assumptions about the origin of scanner variation (26). ComBat implements a multivariate linear mixed effects regression with terms for biological variables and site to model the features of interest; the model parameters are estimated using an empirical Bayes approach. For diffusion tractography, ComBat has already demonstrated higher performance for multiscanner harmonization than other methods such as removal of artificial voxel effect by linear regression (RAVEL) and functional normalization of metrics (26). Unlike a general linear model approach that includes site or scanner as a fixed effect covariate, ComBat demonstrates better outlier robustness to account for small within-scanner sample sizes by borrowing information across features to shrink estimates toward a common mean (27, 28). The multiplicative scanner effects are also corrected by removing heteroscedasticity of model errors across scanners (29). Furthermore, ComBat preserves the variability contributed by true biological effects [e.g., sex and age; (26)]. However, no study has yet examined whether ComBat is suitable for graph theory metrics derived from the structural connectome based on DTI.

Unlike tractography, which yields a final value for each white matter tract, connectome analyses use weighted adjacency matrices to calculate network parameters. In tractography or region-of-interest analyses, multisite harmonization is performed on final metrics [e.g., average fractional anisotropy measures; (26, 30–32)]. However, graph theory analysis takes place after connections in a network have been mapped, mathematically represented as an adjacency matrix, and summarized by the computation of network parameters (33). Two distinct approaches to data harmonization are therefore possible in network analysis: (1) before the calculation of network parameters (i.e., matrix harmonization; harmonization at the level of connectivity weights), or (2) after calculation of network parameters (i.e., parameter harmonization). Identifying the optimal timing of data harmonization during data processing and analysis may influence the harmonization of multisite data, and hence has important implications for the accuracy of conclusions drawn from multisite connectivity studies.

To our knowledge, the performance of ComBat for multiscanner harmonization in studies of network topology and neurological disorders has not been evaluated. Therefore, the present study examined the application of two approaches to data harmonization across sites in a sample of DTI scans from children with mTBI or mild orthopedic injury (OI).

## 2. Methods

### 2.1. Study Design and Procedure

Data were drawn from the Advancing Concussion Assessment in Pediatrics (A-CAP) study (22), a multisite prospective, cohort study with longitudinal follow-up in children [Mean age (range) = 12.37 ± 2.34 years (8.00–16.99 years); 289 (59.7%) Male; see Table 1] with pediatric mTBI (*n* = 313) or mild orthopedic injury (OI; *n* = 171). Briefly, children were recruited within 48 h of injury from five children's hospitals across Canada (32), all of which were members of Pediatric Emergency Research Canada [PERC; (34)], and returned for three post-injury follow-up assessments: post-acute (targeted for 10 days post-injury), 3 and 6 months. Injuries and acute signs and symptoms were assessed during an initial emergency department visit that took place within 48 h post-injury.

**Table 1:** Demographic information for the participants at each site/scanner.

**Site**	**Participants** ***n* (%)**	**Sex** ***n* (%) male**	Age^*^ **M (SD) years**	Group^*^ ***n* (%) mTBI**	DPI^*^ **M (SD)**
Calgary	120 (25)	71 (59)	12.9 (2.2)	83 (69)	8.8 (3.3)
Edmonton	114 (23)	67 (59)	12.5 (2.3)	75 (66)	9.3 (5.1)
Montreal 1	28 (6)	18 (64)	11.4 (2.0)	25 (89)	10.3 (4.2)
Montreal 2	20 (4)	10 (50)	12.5 (2.2)	15 (75)	12.4 (4.8)
Ottawa	57 (12)	30 (53)	11.9 (2.2)	39 (68)	15.8 (4.7)
Vancouver	145 (30)	93 (64)	12.0 (2.4)	76 (52)	11.7 (5.1)
Total	484	289 (60)	12.3 (2.3)	313 (64)	10.9 (5.1)

* *Significant effect of site on age (F = 3.73, p < 0.01), group (χ^2^ = 18.3, p < 0.001), and DPI (F = 21.5, p < 0.001); DPI, days post injury; M, mean; SD, standard deviation*.

The study was conducted with the approval of the research ethics board at each study site. All participants provided written informed assent and parents/guardians provided written informed consent (22). This study examined data from the MRI scans collected during the post-acute visit, as previously described (32, 35).

### 2.2. Diffusion MRI

Eligible participants completed a 3T MRI scan without sedation at the post-acute visit [see (35) for details]. In brief, thirty diffusion-weighted images with different diffusion gradient encoding directions were acquired at b = 900 s/mm^2^, along with five images at b = 0 s/mm^2^, with 2.2 mm isotropic resolution at all sites [General Electric: TR/TE = 6, 12 s/70, 90 ms; Siemens: 6.3, 7.8 s/55, 90 ms; (22)]. Data collected in Montreal was acquired using two different scanners, coded as Montreal 1 and Montreal 2, for a total of 6 scanners (“sites”): Calgary (General Electric), Edmonton (Siemens), Montreal 1 (General Electric), Montreal 2 (Siemens), Ottawa (Siemens), and Vancouver (General Electric).

#### 2.2.1. Quality Assurance

Visual quality checks of all raw images were conducted to identify and exclude scans with structural abnormalities/incidental findings, scanner artifacts (e.g., warping), incomplete acquisition, or not collected using the standardized scan parameters (32). Data that passed the initial quality assessment were subsequently rated for motion by two trained analysts. Discrepancies were resolved through a third reviewer blind to initial ratings. Diffusion-weighted volumes with severe motion artifact were removed, and any scans with > 7 volumes with severe motion artifact were excluded from subsequent analysis (36).

#### 2.2.2. Structural Connectome

Detailed image processing methodology has been previously described (18). Briefly, ExploreDTI (37) was used to preprocess diffusion images, calculate the diffusion tensor, conduct whole brain fiber tractography, and compute an adjacency matrix for each participant. Preprocessing included correction for signal drift (38), eddy currents, subject motion with rotation of the B-matrix (39), and susceptibility distortions (40). A deterministic streamline approach was used for whole brain fiber tractography (randomized seed points; seed and tractography FA threshold = 0.10; step size = 0.50 mm; angle threshold = 30°; step size = 0.5 mm; streamline length 50–500 mm). The resulting whole brain fiber tractography was extracted and used to compute an adjacency matrix for each participant.

The automated anatomical labeling [AAL-90, (41)] template was used to define 90 nodes in native (diffusion) space using functions from open-source software packages in MATLAB R2019a [see (18)]. Fully connected 90 × 90 adjacency matrices were constructed using the average FA of passing fibers among nodes in ExploreDTI for each participant and an absolute threshold of 0.10.

### 2.3. Global Network Metrics and Multisite Harmonization

The following global network parameters were calculated in MATLAB using the GRETNA software toolbox [http://www.nitrc.org/projects/gretna/; (42)]: global efficiency, global clustering coefficient, small worldness, modularity, and density. Network parameters were normalized against 1,000 randomly generated matrices.

**Figure 1:**
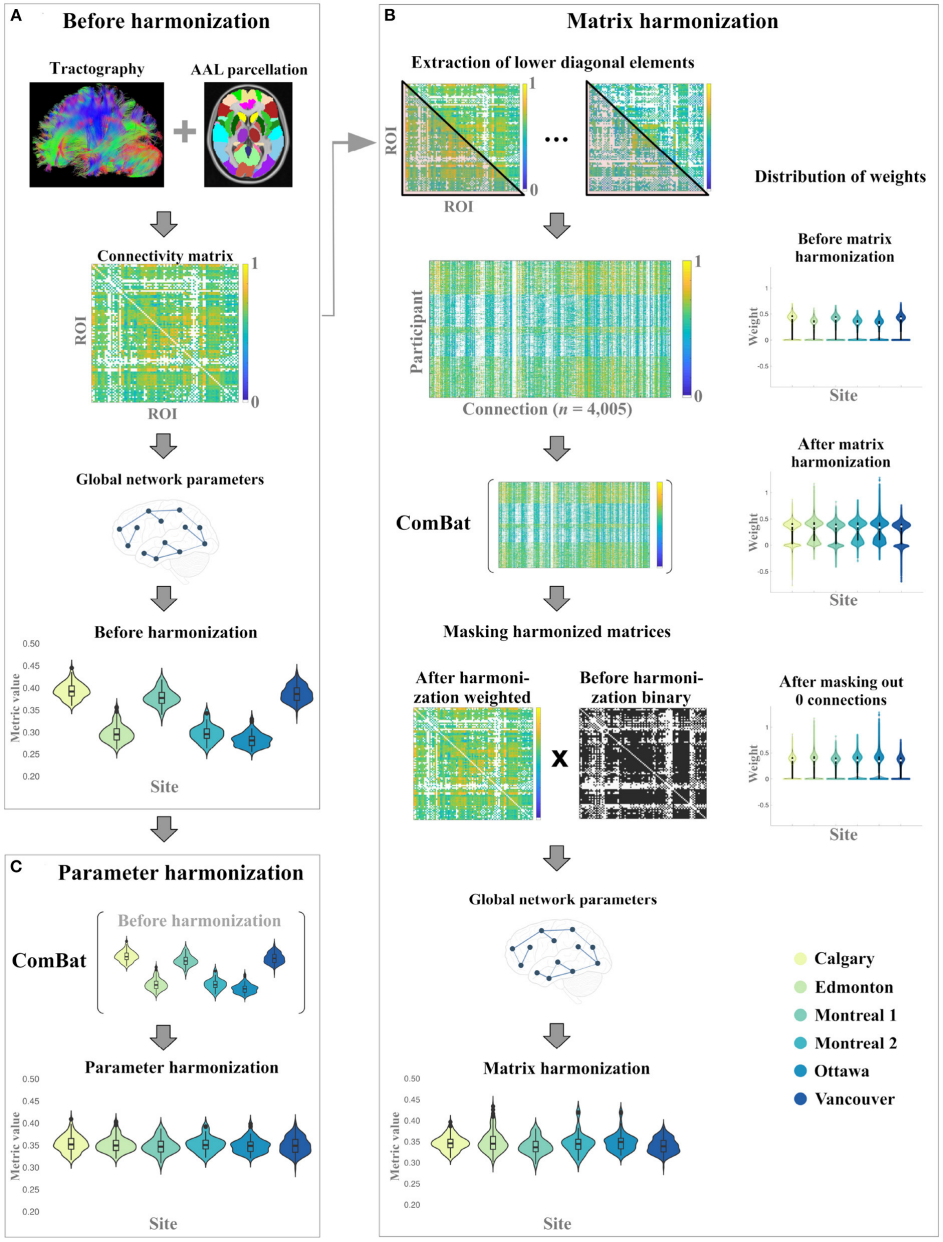
Overall study procedure illustrating the data processing steps for the generation global network parameters **(A)** before harmonization, and the implementation of **(B)** matrix harmonization, and **(C)** parameter harmonization.

Global network parameters were evaluated before harmonization and after two different harmonization approaches: matrix harmonization and parameter harmonization. The steps used for each approach are summarized in Figure 1. For both harmonization approaches, ComBat v1.0.5 (https://github.com/Jfortin1/ComBatHarmonization/tree/master/R) was conducted in R v3.6.3 [(43); https://www.R-project.org/] to harmonize the data for scanner differences. A covariate matrix with group (mTBI/OI), age at injury, and biological sex was included to preserve this variance:


$$\begin{array}{l} mod<-model.matrix\left(\sim injury+age+sex\right) \end{array}$$

#### 2.3.1. Matrix Harmonization

For matrix harmonization, weighted connectivity matrices were harmonized for multiple scanners and global network parameters were calculated using the harmonized connectivity matrix for each participant (see Figure 1). First, the lower diagonal values of each connectivity matrix were extracted to construct a dataframe of 4,005 columns corresponding to node connection pairs among the 90 defined brain regions (nodes), excluding self-connections [i.e., principal diagonal; (n(n-1))/2]. This was done because undirected adjacency matrices are diagonally symmetrical. ComBat was then used to harmonize those extracted values:


$$\begin{array}{l} neuroCombat\left(dat=LowerDiagonal,batch=Site,mod=mod\right) \end{array}$$

After the harmonization of the extracted connectivity weights, the adjusted square and symmetric weighted matrices were reconstructed for each participant and subsequently used to calculate global network parameters. During matrix harmonization, many of the connection weights that were 0 before harmonization (i.e., indicating that no connection existed between two nodes) were transformed to negative values. To correct for this transformation, an additional masking step was applied to reassign negative weights to 0 prior to graph analysis. Specifically, the masking step multiplied the binary connectivity matrix derived before harmonization with the harmonized weighted connectivity matrix for each participant (see Figure 1).

#### 2.3.2. Parameter Harmonization

For parameter harmonization, the raw global network metrics (i.e., calculated before harmonization) were harmonized for multiple scanners using ComBat. Each parameter was harmonized in separate models because the distribution of each parameter is not necessarily related to the distribution of other parameters. The empirical bias estimation option was not applied (i.e., eb = FALSE) during parameter harmonization because each global network parameter was harmonized separately (i.e., number of features < *n*). For each global network metric, the following model was used to harmonize the data:


$$\begin{array}{l} neuroCombat\\ \left(dat=Parameter,batch=Site,mod=mod,eb=FALSE\right) \end{array}$$

### 2.4. Statistical Analysis

Statistical analyses were conducted using R v3.6.3. To evaluate the performance of each harmonization approach (i.e., adjacency matrix or network parameters), the effect of site was examined using separate one-way ANOVA models for each global network parameter. Non-significant scanner effects (*p* > 0.05) were interpreted as a successful removal of variability due to different scanners.

The proportion of significant (*p* < 0.05) *post-hoc* pairwise between-site comparisons was evaluated by calculating the number of significant uncorrected pairwise comparisons across scanners, divided by the total number of possible pairwise comparisons (i.e., *n* = 15). Correction for multiple comparisons was not applied for *post-hoc t*-tests followups, providing a more conservative evaluation of scanner effects.

The within-scanner consistency of the global network metrics before (unharmonized) and after each harmonization approach (matrix harmonization, parameter harmonization) was examined by calculating the intraclass correlation coefficient (ICC), with ICC < 0.50, 0.50 ≤ ICC < 0.75, 0.75 ≤ ICC < 0.90, and ICC ≥ 0.90 indicative of poor, moderate, good, and excellent consistently, respectively (44). Successful harmonization would reduce the effect of site while preserving the within-scanner variability for each parameter observed before harmonization.

To evaluate whether ComBat harmonization preserves biological variability, analysis of covariance (ANCOVA) was used to examine the effect of site, age at injury, sex, and group (mTBI, OI) on each network parameter. Significant effects involving age at injury were further examined using Pearson correlations, which were compared using a back-transformed average Fisher's *Z* procedure for dependent and overlapping correlations (45), as implemented using the cocor package (46). Overlapping correlations were used to conduct the following pairwise comparisons of age correlations: (1) matrix harmonization vs. unharmonized data, (2) parameter harmonization vs. unharmonized data, and (3) parameter harmonization vs. matrix harmonization.

Within-scanner age correlations on the unharmonized data were calculated to provide a reference value for the expected age correlation for each network parameter following harmonization. The reference value was calculated based on the means of within-site age correlations, weighted by the corresponding sample size of each scanner. Weighted means were calculated because sites with a greater number of participants may influence the correlation values to a greater extent than sites with smaller cohorts. Successful preservation of age-related biological variability across all scanners following harmonization would approximate the weighted mean of within-scanner age-correlations. Group differences between mTBI and OI were calculated using *t*-test.

## 3. Results

### 3.1. Presence of Site/Scanner Effects Before Harmonization

**Figure 2:**
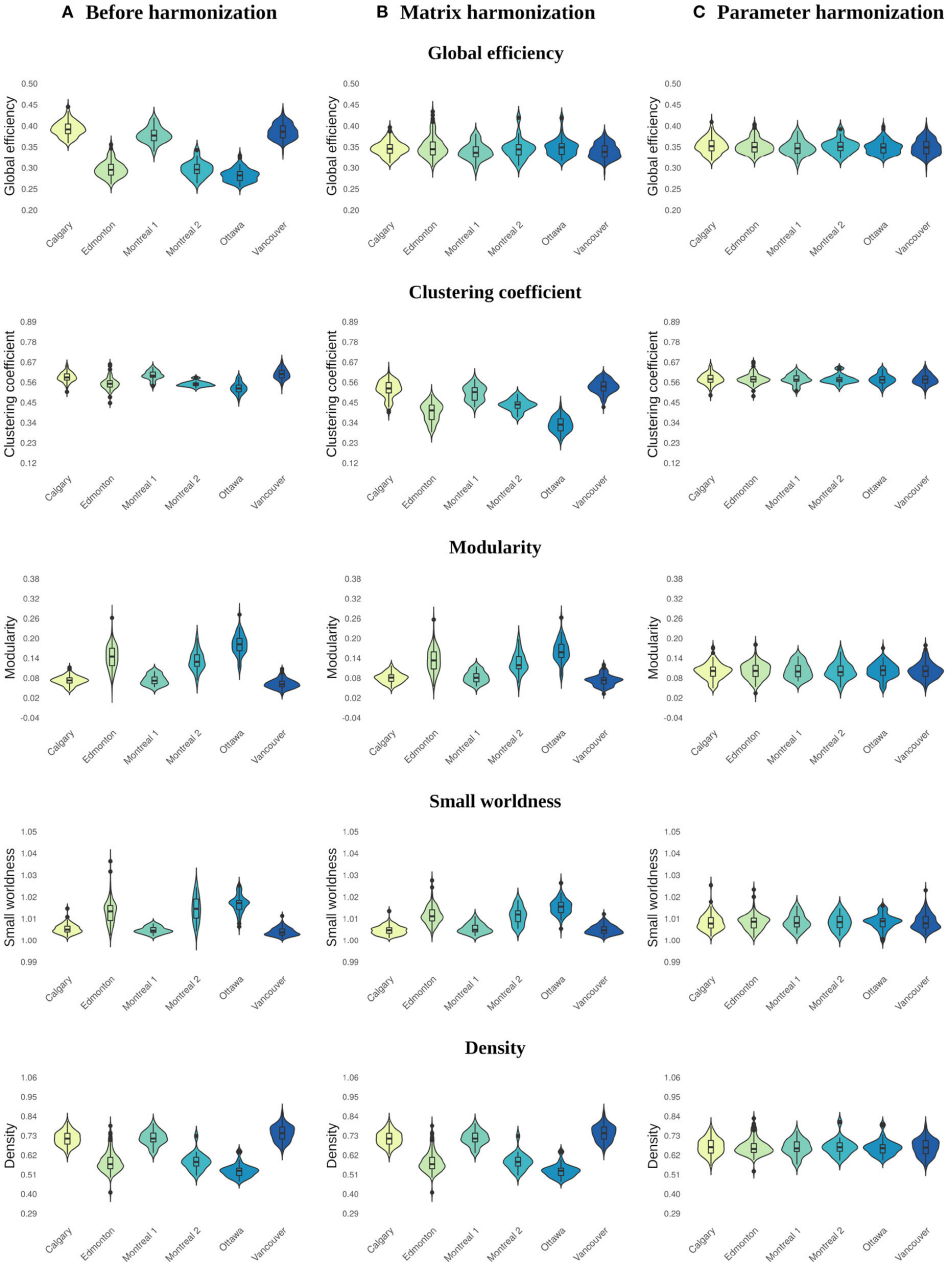
Violin plots illustrating the distribution of values across sites for global network parameters calculated **(A)** before harmonization, after **(B)** matrix, and **(C)** parameter harmonization.

**Figure 3:**
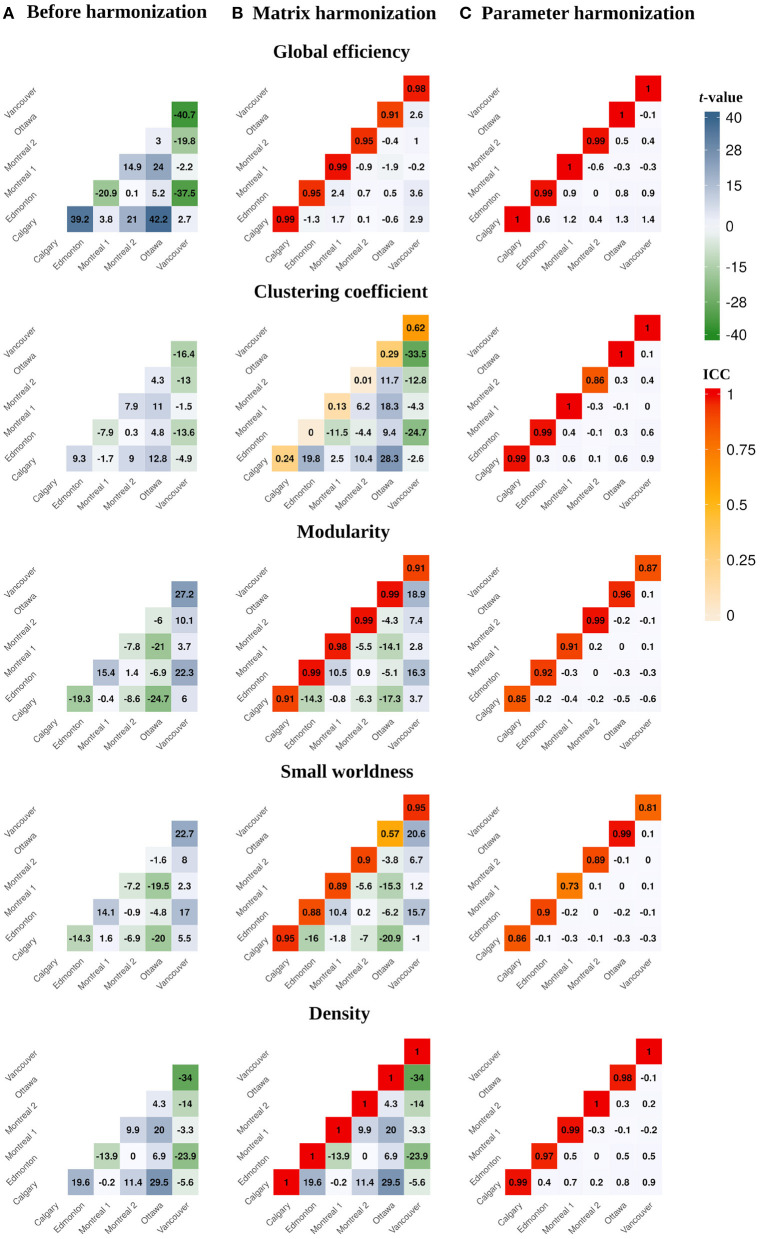
Heatmaps illustrating pairwise between-site differences and *t*-values (lower diagonal) and within-site ICC values (principal diagonal) for the global network parameters calculated **(A)** before harmonization, after **(B)** matrix, and **(C)** parameter harmonization.

Before harmonization, all global network metrics differed by site (see Table 2 and Figures 2A, 3A). The largest site effect was observed for global efficiency [*F*_(5)_ = 651.08, *p* < 0.001], with 14 of 15 (93%) significant between-site comparisons, followed by modularity [*F*_(5)_ = 309.87, *p* < 0.001; 13 (86%) significant between-site comparisons], density [*F*_(5)_ = 286.23, *p* < 0.001; 13 (86%) significant between-site comparisons], small worldness [*F*_(5)_ = 182.93, *p* < 0.001; 12 (80%) significant between-site comparisons], and clustering coefficient [*F*_(5)_ = 86.38, *p* < 0.001; 12 (80%) significant between-site comparisons].

**Table 2:** Results summarizing the overall effect of site on global metrics before harmonization, after matrix harmonization and after parameter harmonization.

**Parameter**	**Before harmonization**			**Matrix harmonization**			**Parameter harmonization**		
	**Overall effect of site**		**No. of pairwise between site differences *p* <0.05 (%)**	**Overall effect of site**		**No. of pairwise between site differences *p* <0.05 (%)**	**Overall effect of site**		**No. of pairwise between site differences *p* <0.05 (%)**
	***F***	**η^2^**		***F***	**η^2^**		***F***	**η^2^**	
Global efficiency	651.08^***^	0.87	14 (93)	3.88^**^	0.04	4 (26)	0.68	<0.01	0 (0)
Clustering coefficient	86.38^***^	0.47	12 (80)	295.44^***^	0.76	15 (100)	0.19	<0.01	0 (0)
Modularity	309.87^***^	0.76	13 (86)	158.06^***^	0.62	13 (86)	0.09	<0.01	0 (0)
Small worldness	182.93^***^	0.66	12 (80)	170.69^***^	0.64	11 (73)	0.02	<0.01	0 (0)
Density	286.23^***^	0.75	13 (86)	286.23^***^	0.75	13 (86)	0.25	<0.01	0 (0)

** *p < 0.001*;

*** *p < 0.0001*.

### 3.2. Matrix Harmonization

Main effects of site remained significant for global network metrics after matrix harmonization (Table 2 and Figures 2B, 3B). However, pairwise site differences were less pervasive after harmonization for global efficiency [*F*_(5)_ = 3.88, *p* < 0.001; 4 (26%) significant between-site comparisons] and small worldness [*F*_(5)_ = 170.69, *p* < 0.001; 11 (73%) significant between-site comparisons], but not for modularity [*F*_(5)_ = 158.06, *p* < 0.001; 13 (86%) significant between-site comparisons], density [*F*_(5)_ = 286.23, *p* < 0.001; 13 (86%) significant between-site comparisons], or clustering coefficient [*F*_(5)_ = 295.44, *p* < 0.001; 15 (100%) significant between-site comparisons].

Within-site consistency of unharmonized and matrix harmonized metrics ranged from poor to excellent (Figure 3B). The highest consistency was observed for density [ICC = 1.00], which is the only parameter that measures the presence of connections but ignores their weights. Global efficiency and modularity also had excellent ICCs [Mean (range) = 0.96 (0.91, 0.99) for both], whereas consistency was good for small worldness [Mean (range) = 0.85 (0.57, 0.95)] and poor for clustering coefficient [Mean (range) = 0.21 (0, 0.62)].

### 3.3. Network Parameter Harmonization

Site was not significantly associated with global network metrics after parameter harmonization (Table 2 and Figures 2C, 3C). The within-site pre-post harmonization ICCs (Figure 3C) were consistently excellent for global efficiency [Mean (range) = 0.99 (0.99, 1)] and density [Mean (range) = 0.98 (0.97, 1)], and good to excellent for clustering coefficient [Mean (range) = 0.97 (0.86, 1)] and modularity [Mean (range) = 0.91 [0.85, 0.99)] and moderate to excellent for small worldness [Mean (range) = 0.86 (0.73, 0.99)].

### 3.4. Relationships Between Network Topology and Age Before and After Harmonization

**Figure 4:**
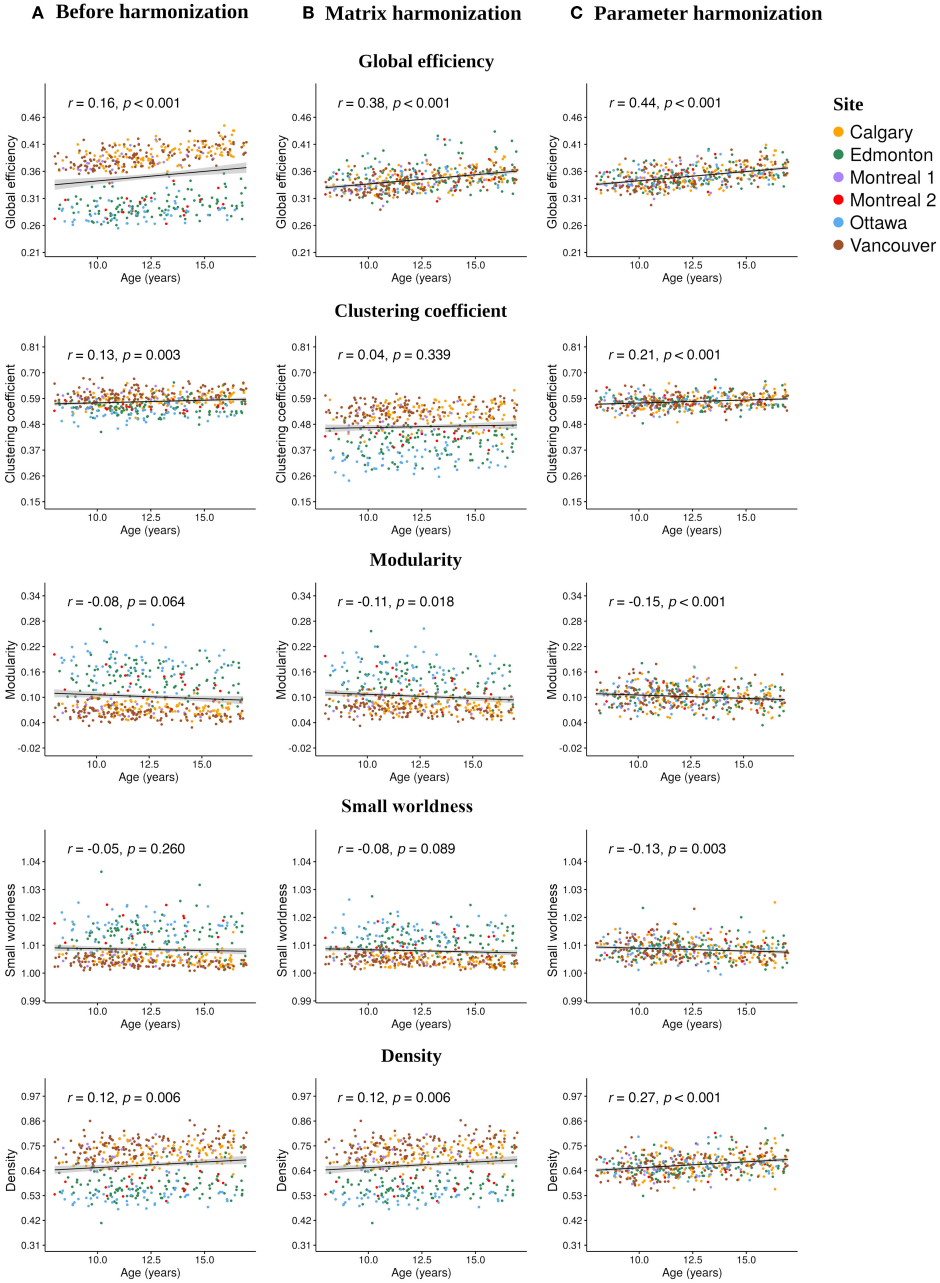
Scatter plots illustrating the Pearson correlations between age at injury and each global network parameter calculated **(A)** before harmonization, after **(B)** matrix, and **(C)** parameter harmonization.

Age significantly correlated with the following unharmonized network parameters (Figure 4A): global efficiency (*r* = 0.16, *p* < 0.001), clustering coefficient (*r* = 0.13, *p* < 0.003) and density (*r* = 0.12, *p* < 0.006). After matrix harmonization (Figure 4B), the correlation between age and global efficiency (*r* = 0.38, *p* < 0.001) was larger than before harmonization (*z* = 3.63, *p* < 0.001). After parameter harmonization, age-correlations increased (Figure 4C) for efficiency (*r* = 0.44, *p* < 0.001), clustering coefficient (*r* = 0.21, *p* < 0.001), and density (*r* = 0.27, *p* < 0.001), although only age correlations for efficiency (*z* = 4.71, *p* < 0.001) and density (*z* = 2.40, *p* < 0.016) were significantly larger compared to the unharmonized data.

No significant correlations with age were observed for modularity and small worldness before harmonization, but modularity significantly correlated with age following matrix harmonization (*r* = −0.11, *p* =0.018), and both parameters showed significant age relationships following parameter harmonization (modularity: *r* = −0.15, *p* < 0.001; small worldness: *r* = −0.13, *p* < 0.003), although the coefficients were not significantly higher compared to the unharmonized data (*p* > 0.05). Clustering coefficient (*z* = 0.26, *p* < 0.008) and density (*z* = 2.40, *p* < 0.016) were significantly stronger following parameter as compared to matrix harmonization. Within-group age correlations are reported in Supplementary Table 1. No significant effects of sex or group were observed for any of the network parameters (see Supplementary Table 2).

## 4. Discussion

The popularity of large, representative datasets from collaborative, multisite research initiatives and structural connectomics has increased in recent years. Previous studies demonstrated that ComBat can control for site (scanner) differences while preserving biological variability (e.g., due to injury group, age, sex) for several MRI modalities (26, 29, 47, 48). This is the first study to validate the use of ComBat for the structural connectome. Here, ComBat was successfully used to harmonize structural connectivity data based on diffusion-weighted MRI across multiple scanners (“sites”). Parameter harmonization reduced the variability associated with different scanners to a greater extent than matrix harmonization, although both approaches reduced site differences in global network metrics. As expected, both harmonization approaches also preserved biological effects of age on network parameters. Moreover, expected age-related associations with global network parameters were stronger after applying parameter as opposed to matrix harmonization. Overall, the results extend the validity of using ComBat harmonization to network parameters derived using diffusion-weighted MRI.

Parameter harmonization showed superior performance for removing scanner effects compared to matrix harmonization. Furthermore, parameter harmonization is more computationally efficient. Matrix harmonization requires a series of steps that involve value translation. Specifically, connectivity weights were deconstructed from the matrices by extracting the lower (or upper) diagonal elements, organized in a high dimensional data frame for harmonization, and reconstructed back in square matrices following harmonization. In some instances, this approach transformed connection weights that were initially 0 (i.e., no connection exists between two nodes in unharmonized data) to negative values, requiring an additional step reassigning these values to zero before graph analysis. Thus, matrix harmonization preserved the location, but not the strength of connections among node pairs. In contrast, parameter harmonization requires only one step. This appears beneficial in preserving the true global properties of the network, as illustrated by the reduced variability of the within-site consistency between the parameter harmonized and unharmonized global network metrics (see Figure 3).

Before harmonization, global efficiency exhibited more robust site effects than other measures, such as clustering coefficient. Matrix harmonization reduced (e.g., global efficiency), introduced (e.g., clustering coefficient), or maintained (e.g., density) site effects compared to the unharmonized data. The variable performance of matrix harmonization across different metrics may indicate that properties of the network other than the pairwise connection strengths are affected by scanner. Except for density, global parameters included in the present analysis encode information about the topology (i.e., location) as well as the weights of connections among distinct brain regions. Density, which reflects the number of connections regardless of their strengths, did not demonstrate differences in the magnitude of site effects following matrix harmonization (see Figures 2, 3), indicating that site differences are present in topological properties of the network beyond the strengths of pairwise connections (e.g., the number or location of connections). In addition, clustering coefficient quantifies segregation across brain regions (i.e., nodes) by counting the occurrence of existing connections between groups of three nodes. Since matrix harmonization does not alter the location of connections, groups of connected nodes maintain their configuration before and after harmonization. Furthermore, the magnitude of scanner effects might differ slightly among connections, and matrix harmonization might differently impact the reciprocal connection strengths across groups of nodes (i.e., it targets pairs of nodes), potentially explaining the variable performance for removing site effects in the case of clustering coefficient (see Figures 3A,B). These topological properties may be better controlled by parameter harmonization, because global parameters already encode this information.

Correlations between age and network parameters were generally larger following both harmonization approaches, but were slightly more robust following parameter harmonization. One exception was the relationship between age and clustering coefficient, which weakened following matrix harmonization. This is in line with the other results, suggesting that matrix harmonization may be problematic for clustering coefficient. The detection of significant age effects following parameter harmonization, even in the absence of significant correlations for the unharmonized data, raises the question of whether additional variability was added to the data during harmonization that might have artificially boosted the relationship between age and network topology. Further analyses suggest this is not the case, because the age correlation following parameter harmonization were closer to the weighted means of within-scanner correlations before harmonization (see Supplementary Table 1). In addition, previous studies show relationships between age and global network topology in typical development (49–51) and children with TBI (19). This indicates that parameter harmonization may better preserve age-related biological variability compared to matrix harmonization and to the unharmonized data, although differences between the two harmonization approaches were small.

The children with mTBI and OI did not differ in any global network metrics before or after each harmonization approach. This was expected given that DTI and NODDI indices of white matter microstructure did not differ between groups previously in this sample (18, 32, 35), and other pediatric samples at similar time points (52). Another study compared a subset of this sample (children recruited at the Calgary site) to typically developing children and also did not find global or regional (nodal) network differences between mTBI and mild OI groups post-acutely, but did find an effect of injury more generally relative to typical development (18).

The current study did not address the effect of data harmonization applied prior to the generation of adjacency matrices, which is an additional possibility to account for the variability across different scanners [e.g., using methods described by (26, 53, 54)]. It has been suggested that connectome generation can be stable across scanners based on the derived network parameters (55). While future studies may consider this, data harmonization prior to connectome construction is increased in complexity, involving additional processing steps. These include warping the data into a common space and deconstructing brain images to build a voxel by participant data frame, which does not allow for the construction of adjacency matrices in native diffusion space. Following voxelwise harmonization, data would need to be reconstructed into subject-specific brain images (i.e., harmonized FA maps), which may impose substantial feasibility challenges due to the high computational complexity and number of additional transformations involved in this process.

There are some limitations to the current work. Weighted connectivity matrices were analyzed in this study; future multisite studies might examine whether differences in binary matrices relate differently to the effects of site. We did not assess the influence of different thresholds on harmonization. In addition, the current study used only one parcellation for the construction of adjacency matrices, and future studies might focus on whether other parcellations are similarly affected by site effects particularly when running matrix harmonization. While most methods use similar preprocessing steps, slight variations in these steps and how they are applied can impact calculated diffusion metrics, and thus may be important to explore in future studies. Data acquisition in the current study included single shell diffusion-weighted data. Multishell acquisition protocols may be differently affected by site effects, which might be addressed in future studies. Lastly, the current study used deterministic tractography, and future analyses might consider testing the effect of harmonization on networks derived using probabilistic tractography, as the two approaches have been shown to differ in terms of within- and between-scanner consistency (55).

## 5. Conclusions

The present paper validates the utility of ComBat harmonization in the context of graph theoretical analysis for structural connectivity derived from DTI. The harmonization of global parameters derived from unharmonized adjacency matrices provided superior performance as compared with the harmonization of connectivity weights for removing between- site differences, preserving the within-site variability and preserving age-related biological variability in the data.

## Data Availability Statement

A dataset with deidentified participant data and a data dictionary will be made available upon reasonable request from any qualified investigator, subject to a signed data access agreement.

## Ethics Statement

The current study was reviewed and approved by each site where data was collected. Written informed consent to participate in this study was provided by the participants' legal guardian/next of kin.

## Author Contributions

AO, AW, AH, KY, and CL: conception and design of the study, acquisition and analysis of data, and drafted significant portion of the manuscript and figures. MB, CB, WC, QD, SF, and RZ: conception and design of the study. All authors contributed to the article and approved the submitted version.

## Funding

Funding provided by the Canadian Institutes of Health Research (FDN143304), Ronald and Irene Ward Chair in Pediatric Brain Injury (KY); Canada Research Chair (CL, AH, and CB); Doctoral Fellowship from the IMT School for Advanced Studies Lucca (AO); Harley N. Hotchkiss-Samuel Weiss and Killam Postdoctoral Fellowship (AW); Alberta Children's Hospital Foundation Professorship in Child Health and Wellness (SF).

## Conflict of Interest

The authors declare that the research was conducted in the absence of any commercial or financial relationships that could be construed as a potential conflict of interest.

## Publisher's Note

All claims expressed in this article are solely those of the authors and do not necessarily represent those of their affiliated organizations, or those of the publisher, the editors and the reviewers. Any product that may be evaluated in this article, or claim that may be made by its manufacturer, is not guaranteed or endorsed by the publisher.
