# Supplementary material for: Multisite Harmonization of Structural DTI Networks in Children: An A-CAP Study
Source: Front Neurol. 2022 Jun 17;13:850642. doi: 10.3389/fneur.2022.850642 (PMC9247315; doi:10.3389/fneur.2022.850642)
Supplement: Supplementary file 1 [file Data_Sheet_1.pdf]

Supplementary Material

1 SUPPLEMENTARY TABLES

Table S1. Pearson correlation coefficients between age and each global parameter within site (scanner) before harmonization.

Site (scanner)	Efficiency	Clustering coefficient	Modularity	Small worldness	Density
Calgary	0.52***	0.39***	-0.29*	-0.26*	0.29*
Edmonton	0.27*	0.10	-0.14	0.03	0.19*
Montreal 1	0.42*	0.12	-0.12	-0.20	0.17
Montreal 2	0.29	-0.14	-0.44	-0.10	0.33
Ottawa	0.24	0.15	-0.05	-0.06	0.13
Vancouver	0.56***	0.22*	-0.22*	-0.30***	0.36***
Weighted mean	0.43	0.21	-0.20	-0.17	0.26

Note. ** $p < 0.001$; *** $p < 0.0001$.

Table S2. Results summarizing the effect of age at injury, sex and group before harmonization, after matrix harmonization and after parameter harmonization (F values).

Parameter	Before harmonization			Matrix harmonization			Parameter harmonization		
	Age	Sex	Group	Age	Sex	Group	Age	Sex	Group
Global efficiency	104.90***	1.09	0.79	76.21***	0.34	1.10	110.80***	1.45	0.62
Clustering coefficient	20.86***	0.26	1.56	1.65	0.14	0.70	21.31***	0.09	1.21
Modularity	12.98***	1.52	0.25	13.59***	2.31	0.01	11.06***	1.42	0.02
Small worldness	3.98*	0.89	0.01	4.40*	0.55	0.04	8.65**	1.06	0.05
Density	34.72***	1.69	0.32	34.72***	1.69	0.32	34.93***	2.29	0.10

Note. * $p < 0.05$; ** $p < 0.001$; *** $p < 0.0001$.
